# Dataset of smart heat and water meter data with accompanying building characteristics

**DOI:** 10.1016/j.dib.2023.109964

**Published:** 2023-12-15

**Authors:** Markus Schaffer, Martin Veit, Anna Marszal-Pomianowska, Martin Frandsen, Michal Zbigniew Pomianowski, Emil Dichmann, Christian Grau Sørensen, Jesper Kragh

**Affiliations:** a Department of the Built Environment, Aalborg University, Aalborg, Denmark; b Department of the Built Environment, Aalborg University, Copenhagen, Denmark

**Keywords:** District heating, Energy use data, Potable water data, Building information

## Abstract

The data presented were sourced from 34,884 commercial smart heat meters and 10,765 commercial smart water meters, spanning a timeframe of up to 5 years (2018–2022). All data primarily originated from single-family houses in Aalborg Municipality, Denmark. Furthermore, comprehensive building characteristics were collected for each building, where available, from the Danish Building and Dwelling Register (BBR) and Energy Performance Certificate (EPC) input data. This effort yielded an extensive pool of up to 86 distinct characteristics per building. All smart meter data were processed employing a well-established methodology, resulting in equidistant hourly data without any erroneous or missing values. The building characteristics derived from the EPCs were additionally filtered using rule sets to improve the data quality. This dataset holds substantial value for researchers involved in the domains of the built environment, district heating, and water sectors.

Specifications Table

**Table:** 

Subject	Civil and Structural Engineering
Specific subject area	Hourly smart heat and water meter data from buildings. Accompanying building characteristics of the buildings.
Data format	Raw Filtered
Type of data	Table Database
Data collection	The hourly smart heat meter data was acquired via commercial smart heat meters (Kamstrup MULTICAL 402, Kamstrup MULTICAL 403, Kamstrup MULTICAL 603) from the local utility company for billing purposes. The hourly smart water meter data was acquired via commercial smart water meters (MULTICAL 21/ flowIQ 210x) from the local utility company for billing purposes. The statistical building characteristics were collected from the Danish Building and Dwelling Register (BBR). The detailed building characteristics were collected from the input data of Danish Energy Performance Certificates (EPCs).
Data source location	Aalborg Municipality, Denmark 57.053, 9.924 Aalborg Forsyning, Danish Building and Dwelling Register, Danish Energy Performance Certificates
Data accessibility	Repository name: AAU VBN - Forefront Research Database Data identification number: https://doi.org/10.5278/7e93e42e-38fc-4d87-ad68-ff1a2d1091aa Direct URL to data: https://vbn.aau.dk/en/datasets/dataset-of-smart-heat-and-water-meter-data-with-accompanying-buil[1] Instructions for accessing these data: Part of the data was originally collected for billing purposes (hourly data from smart heat and water meters) and made available to the authors for scientific purposes via a data use agreement on the legal basis of GDPR article 89. The data were anonymised by the researchers. However, as the data can potentially be deanonymised in combination with the building characteristics through a backward search in the public Danish Building and Dwelling Register, the data are considered personal data subject to the GDPR. Researchers interested in using the data should contact the corresponding author (Anna Marszal-Pomianowska) and are required to complete a joint Data Use Agreement to document that the data sharing is lawful. It should be noted that, for researchers outside the European Union, possible additional requirements apply in accordance with applicable Danish and European law. Once the agreement has been approved, the data which are stored in a PostgreSQL database, can be accessed via an API, which requires authentication via eduGAIN.

## 1 Value of the Data

• This dataset provides an unprecedented amount of data, particularly in conjunction with accompanying building information at a high level of detail. The easy and clearly documented accessibility of the data makes it useful for small- and large-scale research.
• The data can be of great value for research in the built environment, the district heating, and water sectors. It provides countless opportunities for data-driven research and validation of models.
• Within the domain of building-related research, the utility of this dataset becomes evident as it allows for the deepening of current knowledge on the use of heat energy in single-family houses, the refinement of fault detection methods, and the validation of urban building energy models.
• In the field of district heating, the dataset assumes significance, as it facilitates the advancement of research in demand response and load shift, contributing to optimising district heating systems for increased responsiveness and sustainability.
• Together with the building information provided, high-resolution water data could provide valuable insight into the drivers of water use. It has the potential to uncover large-scale consumption patterns, providing a foundation for more effective water resource management strategies.
• The unique combination of high resolution of water and energy data on such a large scale offers new possibilities for novel research possibilities focused on, for example, the separation of energy use for heating and domestic hot water.

## 2 Data Description

**Fig. 1:**
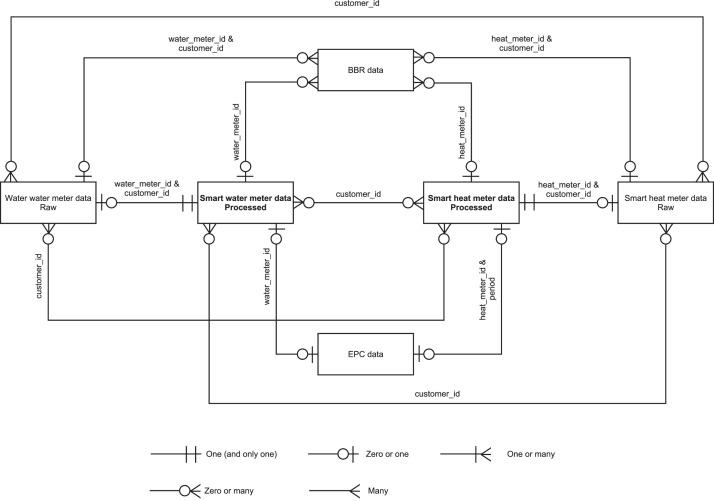
Entity relationship diagram for the database.

**Fig. 2:**
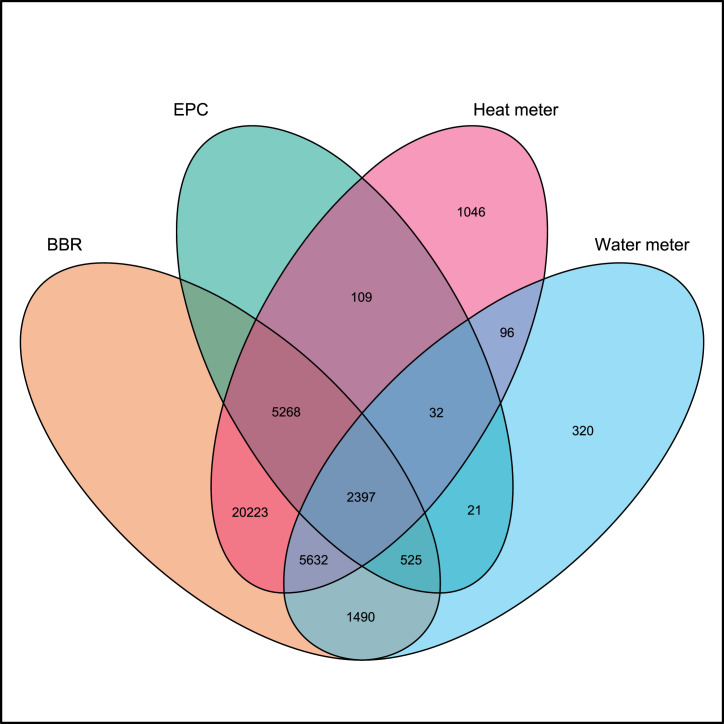
Meter ID and customer ID based number of meters available in the respective group based on the processed data.

The data are structured within six tables in a database. An entity relationship diagram is shown in Fig. 1. All data can be related, which is the core idea of the whole database. The meter ID is unique for all processed data and can be used as an identifier. For the raw smart meter data, it should be noted that there may be meters that are incorrectly assigned to two customers, so the uniqueness of the ID is not guaranteed for the raw data. The customer ID can be used to link Smart Heat Meter (SHM) and Smart Water Meter (SWM) data. It should be noted that a customer can have one or more meters. For this reason, there may be duplicate entries in the Danish Building and Dwelling Register (BBR) data, differing only in the meter ID, e.g., if a customer has one SHM but two SWM, then there are two entries, identical except for the SWM ID (both entries have the same SHM ID). As EPCs are only valid for 10 years and due to the established validity criteria as outlined in Section 3.4, the EPC data have a dependency on the data period. Due to this, there may be several identical entries for the same building, e.g., one for the SWM data, one for the SHM data, or several for the SHM data if the SHM data have several periods. Fig. 2 gives an overview of the number of meters for which the respective data (processed data for SHM and SWH data) are available in the database. In the following, each table is described separately.

### 2.1 Hourly smart heat meter data

#### 2.1.1 Raw data

This table contains the data as collected by the SHMs installed in the respective buildings in the Municipality of Aalborg, Denmark. An overview of all columns included in this dataset is given in Table 1. The data span from the beginning of 2018 to the end of 2022 (with different lengths for each building) and contain data from a total of 34884 SHMs (9.46e+08 rows). Data from a building may not be complete, that is, a building may have data from 2018 and 2020 but no data from 2019. The data have not been processed in any way other than by eliminating redundant columns of units of measurement to reduce the amount of storage space required. As the data are not processed, they are not exactly hourly, as the SHMs have a temporal accuracy of ±30 min around the full hour. In addition, the original data were delivered to the researchers with a timestamp in local time (CET/CEST) but without any time zone information. Consequently, for readings between 2 and 3 o'clock on the day where summertime ends, and thus the hour between 2 and 3 o'clock exists twice, once in summertime (CEST) and once in standard time (CET), it cannot be distinguished if these readings originate from CEST or CET. The data contain missing values due to errors in the transmission infrastructure used to collect the data.

**Table 1:** Description of the raw and processed smart heat meter (SHM) data.

Column name	Raw data	Processed data	Description	Unit
customer_id	✓	✓	Hashed customer ID. Unique for every customer of the utility company.	-
heat_meter_id	✓	✓	Hashed meter ID. Unique for every SHM (guaranteed unique only in the processed data).	-
period		✓	As a meter can have data for nonconsecutive years, the period indicates if the data of one meter is continuous or from two or more separated years. A period is thereby an integer ranging from 1 to n.	-
reading_time	✓		Original reading time of the SHM given in local time (CET). It is to be noted, the time is saved in the database correctly parsed with the time zone. However, originally, the time was supplied without a time zone. Thus, the time can be incorrect when the daylight-saving time ends.	-
time_rounded	✓	✓	Equidistant timesteps as a result of the data processing given in local time (CET/CEST).	-
heat_energy_kwh	✓	✓	Cumulative heat energy deposited. Raw values are rounded down to integer values.	kWh
heat_energy_kwh_demand		✓	Calculated hourly energy use.	kWh
heat_energy_kwh_spms		✓	By SPMS [2] treated energy use data. This reduces the rounding error introduced by the rounding down of the original data.	kWh
volume_m3	✓	✓	The cumulative volume of district heating water passed through the SHM - measured at the supply. Raw values are rounded down to 0.01.	m³
volume_m3_demand		✓	Calculated hourly volume use.	m³
flow_x_temp_supply_m3C	✓	✓	The cumulative volume flow of the supply multiplied by the supply temperature. Raw values are rounded down to integer values.	m³°C
flow_x_temp_supply_m3C_demand		✓	The demand value of the volume flow of the supply multiplied by the supply temperature.	m³°C
flow_x_temp_return_m3C	✓	✓	The cumulative volume flow of the supply multiplied by the return temperature. Raw values are rounded down to integer values.	m³°C
flow_x_temp_return_m3C_demand		✓	The demand value of the volume flow of the supply multiplied by the return temperature.	m³°C
was_missing		✓	Binary column indicating if a value was imputed.	
supply_temp_C	✓		Instantaneous supply temperature at the time of reading (reading_time).	°C
return_temp_C	✓		Instantaneous return temperature at the time of reading (reading_time).	°C
supply_flow_m3	✓		Instantaneous supply flow at the time of reading (reading_time).	m³/h
time_counter_h	✓		Number of hours the SHM has been in operation.	h
heat_power_kw	✓		Current deposited heating power at the time of reading (reading_time) – not recorded for all meter types.	kW
meter_type	✓		SHM model type.	-

#### 2.1.2 Processed data

**Fig. 3:**
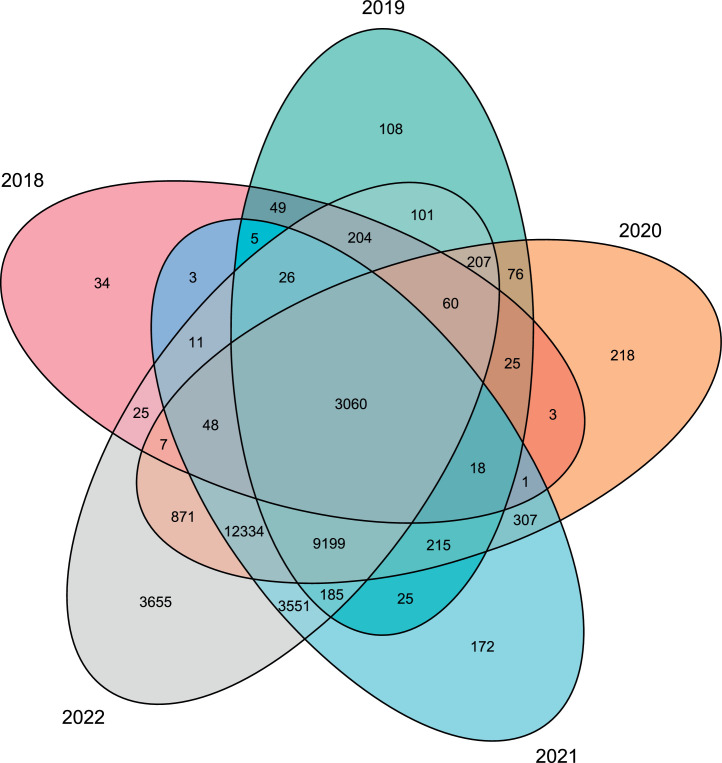
Number of processed SHMs available in the different years of the data period.

The processed data table contains the processed data from the SHMs. It contains data from 34795 SHMs (9.33e+08 rows), and an overview of all available columns is given in Table 1. These data are equidistant, have no erroneous values (in terms of transmission errors or incorrect meter assignment), and missing values have been imputed. The processing used is described in detail in Section 3.1. Fig. 3 shows the number of processed SHMs available for the different years of the data period.

### 2.2 Smart water meter data

#### 2.2.1 Raw data

This table contains the data as collected via the SWMs installed in the respective buildings in Aalborg Municipality, Denmark. An overview of all columns included in this dataset is given in Table 2. The dataset covers the period from the beginning of May 2021 to the end of 2022 (with different lengths for each building) and contains in total data from 10765 SWMs (7.19e+07 rows). The data have not been processed in any way other than by removing redundant columns containing units of measurement to reduce the amount of storage required. As the data have not been processed, they are not exactly hourly as the SWMs have a time accuracy of ±30 min around the full hour. The data have been supplied with UTC timestamps, so unlike the SHM data, the timestamp is always correct.

**Table 2:** Description of the raw and processed smart water meter (SWM) data.

Column name	Raw data	Processed data	Description	Resolution
customer_id	✓	✓	Hashed customer ID. Unique for every customer of the utility company. One customer can have multiple SWMs.	-
water_meter_id	✓	✓	Hashed meter ID. Unique for every SHM (guaranteed unique only in the processed data).	-
reading_time	✓		Original reading time of the SWM given in local time (CET/CEST). In contrast to the smart heat meter data, these data were originally saved in UTC, and thus there is no issue with the daylight-saving time.	-
time_rounded	✓	✓	Equidistant timesteps as a result of the data processing given in local time (CET/CEST).	-
water_volume_m3	✓	✓	Cumulative water volume. Raw values are rounded down to 0.001 (1L).	m^3^
water_volume_demand_m3h		✓	Hourly usage of water.	m^3^
was_missing		✓	Binary column indicating if a value was imputed.	-

#### 2.2.2 Processed data

The processed data table contains the processed data from the SWMs. It contains data from 10,510 SWMs (7.04e+07 rows), and an overview of all available columns is given in Table 2. These data are equidistant, have no erroneous values (in terms of transmission errors or incorrect meter assignment), and missing ones have been imputed. The processing used is described in detail in Section 3.2.

### 2.3 Statistical building characteristics (BBR)

For each building for which either SHM or SWM data are available in this dataset, the corresponding data from the BBR have been collected where possible. This publicly available database in Denmark contains information on every building in Denmark and is operated by the Danish Customs and Tax Administration. An overview of the available columns is given in Table 3. The process of collecting the data is described in Section 3.3.

**Table 3:** Description of data derived from the Danish building and dwelling register.

Column name	Type	Description
heat_meter_id	hash	Unique hashed meter ID, which functions as the key to link the data to the smart heat meter data.
water_meter_id	hash	Unique hashed meter ID, which functions as the key to link the data to the smart water meter data.
unit_type_code	nominal	Type of unit, such as a single-family house, apartment, etc.
unit_housing_type_code	nominal	Information if the unit is a residential apartment, mixed-used, single room, etc.
unit_total_area	float	Total area of the unit.
unit_residential_area	float	Total residential area of the unit.
unit_business_area	float	Total business area of the unit.
unit_nr_room	integer	Number of rooms in the unit.
unit_toilet_pos_code	nominal	Information if the toilet is positioned inside the unit or outside the unit.
unit_bath_pos_code	nominal	Information if a bathroom exists and if the bathroom is positioned inside the unit or outside the unit.
unit_kitchen_pos_code	nominal	Information if a kitchen exists and if the kitchen is positioned inside the unit or outside the unit.
unit_energy_code	nominal	Information about which voltage of electricity is available in the unit and if gas is available.
unit_nr_business_room	integer	Number of business rooms per unit.
unit_other_area	float	Total area which is neither business nor residential.
unit_rent_status_code	nominal	Information if the unit is used by the owner or rented out.
unit_heating_code	nominal	Type of heating available in the unit.
unit_heating_carrier_code	nominal	Type of heating carrier used for heating the unit.
unit_sup_heating_code	nominal	Type of supplementary heating available in the unit.
unit_nr_toilet	integer	Number of water-flushed toilets in the unit.
unit_nr_bathroom	integer	Number of bathrooms, defined as a room with a shower and/or bathtub, in the unit.
bldg_type_code	nominal	Same as unit_type_code but on the building level.
bldg_nr_units_w_kitchen	integer	Number of units with kitchen in the building.
bldg_nr_units_wo_kitchen	integer	Number of units without a kitchen in the building.
bldg_constrcution_year	integer	Construction year of building.
bldg_conversion_year	integer	Year of renovation of the building.
bldg_ext_wall_mat_code	nominal	Type of external façade cladding.
bldg_roof_mat_code	nominal	Type of roof cladding material.
bldg_sup_ext_wall_mat_code	nominal	Type of supplementary external façade cladding.
bldg_sup_roof_mat_code	nominal	Type of supplementary roof cladding material.
bldg_total_area	float	Building total area.
bldg_residential_area	float	Building residential area.
bldg_business_area	float	Building business area.
bldg_developed_area	float	Developed area of the building.
bldg_nr_floor	integer	Number of floors in the building.
bldg_floor_code	nominal	Information about the floors, e.g., if the building has double high storeys or deviating floors.
bldg_heating_code	nominal	Same as unit_heating_code but on the building level.
bldg_heating_carrier_code	nominal	Same as unit_heating_carrier_code but on the building level.
bldg_sup_heating_code	nominal	Same as unit_sup_heating_code but on the building level.
bbr_resolution	nominal	Information on whether the address could be attributed to a unit or a building. If the address could only be linked to a building, information about the unit are missing.

### 2.4 Detailed building characteristics (EPC)

For each building for which either SHM or SWM data are available in this dataset, the input data from the corresponding Energy Performance Certificate (EPC), if available, were collected and processed from the EPC database developed by Brøgger and Wittchen, 2016 [3] and hosted at Aalborg University. An overview of the available columns is given in Table 4. The processing used to derive the data is described in Section 3.4.

**Table 4:** Description of data derived from the Danish energy performance certificate.

	Column name	Unit/Type	Description
General information	heat_meter_id	hash	Unique meter ID, which functions as the key to link the data to the smart heat meter data.
	water_meter_id	hash	Unique meter ID, which functions as the key to link the data to the smart water meter data.
	period	integer	As a meter can have data for non-consecutive years, the period indicates if the data of one meter is continuous or from two or more separated years. A period is thereby an integer ranging from 1 to n.
	valid_from	DateTime	Start date of EPC.
	valid_to	DateTime	End date of EPC.
General building characteristics	bbr_use_code	nominal	Use code as defined in the Danish Building and Dwelling Register (translated).
	total_heated_floor_area	m²	The total heated floor area of the building. It is to be noted that this is 0m² for a considerable number of buildings.
	heated_commercial_area	m²	Commercial area of the building.
	height	m	Room height.
	floor_count	-	Number of floors of the building.
	heat_capacity	Wh/(m^2^K)	Simplified heat capacity of the building according to DS/INF 418-2:2014 (or an earlier version if the data is based on an EPC from before 2014) per unit gross area.
Opaque envelope	opaque_heatloss_kelvin	W/K	Total heat loss through the opaque envelope per Kelvin calculated as follows: $\;\sum_{n=1}^{i}{\mathrm{area}}_{n}\times u\;{\mathrm{value}}_{n}\times \mathrm{temperature}\;{\mathrm{factor}}_{n}$ 1 The temperature factor is a fraction between 0 and 1, used to account for the fact that the outside of a building component may face a different temperature than the outside air temperature or that inside of a component can face a different temperature than the room temperature. This is 1.0 for the 'standard' case and 0.7 is commonly used for cases such as a ground deck without underfloor heating or exterior walls of the basement deeper than 2 metres.
Opaque envelope	opaque_heatloss_total	W	Total heat losses through the opaque envelope, taking the dimensioning temperature into account, were calculated as follows: $\;\sum_{n\;=\;1}^{i}{\mathrm{area}}_{n}\times u\;{\mathrm{value}}_{n}\times \mathrm{temperature}\;{\mathrm{factor}}_{n}\;\times \;\left(\text{dim}.\;\text{int}.\;\text{temp}.\;-\;\text{dim}.\;\text{ext}.\;\text{temp}.\right)\;$ 2 For an explanation of the temperature factor see Equation 1. The dimensioning temperatures are thereby calculated based on the Danish standard DS 418:2011. Standard values are thereby 20°C for the interior, 30°C interior temperature for a floor with floor heating, -12°C for the exterior, and 10°C for exterior elements against soil deeper than 2m.
Window north	window_heatloss_north_kelvin	W/K	Heat losses per Kelvin through all windows facing north (orientation > 315° OR orientation <= 45°), calculated as: $\;\sum_{n\;=\;1}^{i}nr\;of\;windows_{n}\;\times \;area_{n}\times u\;value_{n}\times temperature\;factor_{n}$ 3 For an explanation of the temperature factor see Equation 1.
	window_heatloss_north_total	W	Total heat losses through all windows facing north (orientation > 315° OR orientation <= 45°), taking the dimensioning temperature into account is calculated as: $\;\sum_{n\;=\;1}^{i}nr\;of\;windows_{n}\;\times \;area_{n}\times u\;value_{n}\times temperature\;factor_{n}\;\times \;\left(dim.\;int.\;temp.\;-\;dim.\;ext.\;temp.\right)\;$ 4 For an explanation of the temperature factor see Equation 1. For an explanation of the dimensioning temperatures see Eq. 2.
	window_solar_north	m²	Total solar factor of all windows facing north (orientation > 315° OR orientation <= 45°), calculated as: $\;\sum_{n\;=\;1}^{i}nr\;of\;windows_{n}\times area_{n}\times g\;value_{n}\times glass\;share_{n}\times \mathrm{shading}\;\mathrm{factor}$ 5 Whereby the shading factor was calculated from the angles to the shading objects of each window based on the simplified method stated in [4]. For objects shading from the side as well as overhang, an infinite height and length were assumed. It is to be noted that the shading from the wall thickness could not be considered as the simplified method is based on the wall thickness, which is not an input for EPCs.
Window east	window_heatloss_east_kelvin	W/K	Heat losses per Kelvin through all windows facing east (orientation > 45° AND orientation <= 135°), calculated as stated in Eq. 3.
	window_heatloss_east_total	W	Total heat loss through all windows facing east (orientation > 45° AND orientation <= 135°), taking the dimensioning temperature into account, is calculated as stated in Eq. 4.
	window_solar_east	m²	Total solar factor of all windows facing east (orientation > 45° AND orientation <= 135°), calculated as stated in Eq. 5.
Window south	window_heatloss_south_kelvin	W/K	Heat losses per Kelvin through all windows facing south (orientation > 135° AND orientation <= 225°), calculated as stated in Eq. 3.
	window_heatloss_south_total	W	Total heat loss through all windows facing east (orientation > 135° AND orientation <= 225°), taking the dimensioning temperature into account, is calculated as stated in Eq. 4.
	window_solar_south	m²	Total solar factor of all windows facing south (orientation > 135° AND orientation <= 225°), calculated as stated in Eq. 5.
Window west	window_heatloss_west_kelvin	W/K	Heat losses per Kelvin through all windows facing west (orientation > 225° AND orientation <= 315°), calculated as stated in Eq. 3.
	window_heatloss_west_total	W	Total heat loss through all windows facing east (orientation > 225° AND orientation <= 315°), taking the dimensioning temperature into account, is calculated as stated in Eq. 4.
	window_solar_west	m²	Total solar factor of all windows facing west (orientation > 225° AND orientation <= 315°), calculated as stated in Eq. 5.
Skylight	skylight_heatloss_kelvin	W/K	Heat losses per Kelvin through all skylights were calculated as stated in Eq. 3.
	skylight_heatloss_total	W	Total heat loss through all skylights, taking the dimensioning temperature into account, is calculated as stated in Eq. 4.
	skylight_solar	m²	The total solar factor of all skylights was calculated as stated in Eq. 5.
Thermal bridge	thermal_bridge_kelvin	W/K	Total heat losses through thermal bridges were calculated as follows: $\;\sum_{n=1}^{i}length_{n}\times \psi \;value_{n}\times temperature\;factor_{n}$ 6
	thermal_bridge_total	W	Total heat losses through thermal bridges, taking the dimensioning temperature into account, calculated as follows: $\;\sum_{n\;=\;1}^{i}\begin{array}{c} length_{n}\times \psi \;value_{n}\times temperature\;factor_{n}\\ \;\times \;(dim.\;int.\;temp.\;-\;dim.\;ext.\;temp.)\; \end{array}$ 7
Domestic hot water tank	dhw_tank_volume	L	Total domestic hot water tank volume calculated as: $\;\sum_{n\;=\;1}^{i}nr\;of\;tanks_{n}\times volume_{n}$ 8 It is, however, to be noted that the Danish EPC calculation method is insensitive to the tank volume. For this reason, many buildings have a total share of domestic hot water covered by the domestic hot water tank larger than zero with a 0L tank volume.
	dhw_tank_heat_loss	W/K	Total heat losses from domestic hot water tanks were calculated as follows: $\;\sum_{n\;=\;1}^{i}nr\;of\;tanks_{n}\;\times heat\;loss_{n}\times temperature\;factor_{n}$ 9
Domestic hot water tank	dhw_tank_sup_temp	°C	The required supply flow temperature from the central heating system to the domestic hot water tank was calculated as follows: $\;\frac{\sum_{n\;=\;1}^{i}supply\;temperature_{n}\;}{n}\;$ 10 Due to the above-mentioned fact that a large share of EPCs have a tank volume of 0l, the tank volume is not considered for averaging.
	dhw_tank_share	-	The total share of domestic hot water covered by the domestic hot water tanks. Calculated as: $\;\sum_{n=1}^{i}\mathrm{share}\;\mathrm{of}\;{\mathrm{consumption}}_{n}$ 11
	dhw_tank_el_support_code	nominal	A factor indicating whether the domestic hot water tank has electrical heating. The factor has four levels: • *None*: no electrical heating • *Always*: Electric heating is always available • *Summer:* Electric heating is only available in summer available • *No tank* The value was derived based on the maximum number of tanks with the respective electric heating possibility. (The volume could not be used, due to the above problem, that many EPCs have erroneously a 0l tank.)
Domestic hot water tank	dhw_average_consumption	L/year	Domestic hot water demand calculated as: heateddwellingarea×averageDHWuse 12 Default values for DHW are 250L/(m^2^ year) in residential buildings and 100L/(m2 year) for non-residential buildings.
	dhw_temperature	°C	Domestic hot water temperature.
	dhw_pipes	W/K	Total heat losses through DHW pipes were calculated as follows: $\;\sum_{n\;=\;1}^{i}length_{n}\times \text{heat}\;\text{los}s_{n}\times temperature\;factor_{n}$ 13
Internal gains	gains_people	W	Total heat gains from occupants, calculated as follows: $\;\sum_{n\;=\;1}^{i}area_{n\;}\times occ\;heat\;gains\;\text{per}\;\text{are}a_{n}$ 14 Default values for internal heat gains from occupants are 1.5 W/m^2^ but at maximum 360 W for residential buildings and 4 W/m^2^ for non-residential buildings.
	gains_device	W	Total heat gains from appliances inside usage hours, calculated as follows: $\;\sum_{n\;=\;1}^{i}area_{n\;}\times appliances\;heat\;gains\;per\;area_{n}$ 15
	gains_device_outside	W	Total heat gains from appliances outside usage hours were calculated as stated in Eq. 15.
Heating system	heating_supply_temp	°C	Supply temperature of the heat distribution system.
	heating_return_temp	°C	Return temperature of the heat distribution system.
	heating_pipes	W/K	Total heat losses through heating pipes, calculated as: $\;\sum_{n\;=\;1}^{i}length_{n}\times \text{heat}\;\text{los}s_{n}\times temperature\;factor_{n}$ 16
	heating_type_code	nominal	Plant type: • 1: Single-circuit system • 2: Double circuit system (or parts of the installation are single circuit, and these are equipped with local mixing devices)
Ventilation winter	vent_nat_winter	L/s	Total natural ventilation in winter, calculated as follows: $\;\sum_{n\;=\;1}^{i}area_{n\;}\times \mathrm{ventilation}\;\mathrm{flow}\;\mathrm{per}\;{\mathrm{area}}_{n}\times \text{usage}\;\text{facto}r_{n}$ 17
	vent_mech_winter	L/s	Total mechanical ventilation in winter, calculated as follows: $\;\sum_{n=1}^{i}{\mathrm{area}}_{n}\times \mathrm{ventilation}\;\mathrm{flow}\;\mathrm{per}\;{\mathrm{area}}_{n}\times \mathrm{usage}\;{\mathrm{factor}}_{n}$ $\;\times \;\mathrm{temperature}\;\;{\mathrm{efficiency}}_{n}$ 18 Temperature efficiency refers to the efficiency of the heat recovery.
Ventilation system	vent_inlet_temperature_code	nominal	Categorisation of ventilation, heat recovery and heating coil, based on the maximum vent_mech_winter for the first three categories. If vent_mech_winter is zero, “Type 4” is selected. • Type 1 = ventilation system with temperature-controlled heat recovery (and temperature-controlled heating coil) • Type 2 = ventilation system with NOT temperature-controlled heat recovery and temperature-controlled heating coil • Type 3 = ventilation system with NOT temperature-controlled heat recovery and NO (temperature-controlled) heating coil • Type 4 = no mechanical ventilation system
Ventilation summer	vent_nat_summer	L/s	Total natural ventilation in summer was calculated as stated in Equation 17.
	vent_mech_summer	L/s	Total mechanical ventilation in summer calculated as: $\;\sum_{n\;=\;1}^{i}area_{n\;}\times ventilation\;flow\;per\;area_{n}\times \;usage\;factor_{n}$ 19
Solar plant	solar_plant_type_code	nominal	Type of solar plant: • None = No solar plant (respectively solar plant with 0m^2^ area) • UtilityWater = only for domestic hot water • RoomHeating = only for room heating • Combined = Combined for room heating and domestic hot water
	solar_plant_area	m^2^	Area of the solar plant
Heat pump	heatpump_type_code	nominal	Types of solar plant: • None = No heat pump (respectively heat pump with 0 area fraction) • RoomHeating = only for room heating • UtilityWater = only for domestic hot water • Combined = One heat pump combined for room heating and domestic hot water • Duo = Two heat pumps, one for room heating and one for domestic hot water
	heatpump_area_fraction	-	Proportion of the total heated floor area of the building covered by the heat pump. If heat pumps supply heat to the ventilation system's supply air, a negative number indicates that there is also other heating in the rooms.

## 3 Experimental Design, Materials and Methods

### 3.1 Smart heat meter data processing

The SHM data were obtained by the authors from the local utility company as .csv files. As mentioned above, the readings were provided in local time (CET/CEST) without any time zone information. As the dataset is similar to the one described in detail by Schaffer et al. [5], a similar cleaning and imputation framework was applied to obtain equidistant data without erroneous or missing values. The only difference from the framework described in Schaffer et al. [5] is that, due to the long data period and the higher uncertainty in data quality, it was tested that there were at least 8584 h of data per year and per smart meter (approximately 2 % of missing data). If this threshold was exceeded, only the year in question was excluded. Thus, an SHM may have data in nonconsecutive years in the processed data. Consequently, these data sequences can be considered as separate data. For this reason, the period column (Table 1) has been introduced. This column, starting with one, indicates whether the SHM data are from a different sequence, i.e., if an SHM has data in 2018 and 2020–2022 but no data in 2019, the period column is 1 for all data in 2018 and 2 for all data in 2020–2022.

In addition to this basic data treatment, the SPMS method developed by Schaffer et al. [2] was applied to energy use. SPMS was developed to reduce the error introduced by rounding the raw cumulative energy data to integer values. The result of this process is available as a separate column (heat_energy_kwh_spms) in the processed data (Table 1).

### 3.2 Smart water meter data processing

The authors obtained the SWM data from the local utility company as .csv files. The data were provided with readings in UTC. Given the same nature of the data (cumulative and approximately hourly), the same cleaning and imputation framework as for the SHM data was used to process the SWM data. However, given the varying data period, the threshold for missing values was set at 2% for each SWM individually, based on the first and last recorded value, to account for the different lengths of the datasets. SWMs with more than 2% missing values were excluded.

### 3.3 BBR data processing

The address was the only customer information provided by the utility company to link SHM and SWH data to a building/unit. It was unclear whether the address referred to a unit (e.g., an apartment) or a building (e.g., an apartment building). The address was used to retrieve the building characteristics from the BBR database. To prevent incorrect information from influencing the retrieval of building characteristics, the address information provided was treated with the Address Cleaning API, which is part of the Danish Address Web API (DAWA) [6]. This API can translate unstructured addresses with possible misspellings into official addresses. In addition to the address information, the API returns the certainty of the match expressed in three levels: A - identical match, B - certain match, and C - uncertain match. Only results with a confidence of A or B were considered valid. As the address cleaning API distinguishes between unit and building addresses, all addresses were initially treated as unit addresses, and only addresses with a certainty of C were subsequently treated as building addresses. Addresses for which neither a unit nor a building address could be found with high confidence (level A or B) were excluded.

The BBR information was obtained through Denmark's Address Web API (DAWA) [6]. Information about a unit and its building could be obtained directly through the API. For the SHMs where only a building address was available, the 'access address id' had to be retrieved via the address before information about the building could be obtained. In both cases, more than one BBR record may be obtained, for example, if two or more units/buildings have the same address. In order to allow for a data structure where an SHM can be linked to zero or one BBR record, cases where more than one record was obtained were considered invalid and consequently not included in the database. All nominal values were translated into human-understandable terms in English.

As the main objective was to establish essential building characteristics for as many SHMs as possible, only mandatory BBR information was considered for the dataset. The building owner must provide this mandatory information and it is, therefore, subject to uncertainty. However, the data quality has recently been investigated [7] and it was concluded that the overall quality of the data is high and that the data quality has improved from 2000 to 2013.

### 3.4 EPC data processing

To link the available EPC data from the EPC database developed by Brøgger and Wittchen, 2016 [3] and hosted at Aalborg University with the SHM and SWM data, the same 'cleaned' addresses as for the BBR data (Section 3.3) were used. Given the sheer amount of information available in the EPCs, it was decided to focus mainly on data from five aspects:

• Building envelope
• Domestic hot water (DHW)
• Ventilation
• Heating
• Internal heat gains

The data quality of the Danish EPC has been heavily criticised in the past, as random checks have revealed errors in 20–30 % of all EPCs [8]. For this reason, the cleaning framework developed by Brøgger [8] was applied. However, this framework was originally developed for the purpose of energy modelling of the building stock. Therefore, some criteria have been adapted, and some have been added to better fit the purpose of this dataset. All quality assurance criteria used can be found in the dataset repository [1].

After the cleaning step, the information obtained was aggregated to obtain the same building characteristics for each building where the information was available. The resulting columns, including a description of how they were calculated, are shown in Table 4. Only results where an EPC record could be clearly linked to one building were considered. Furthermore, only valid EPCs were considered. Validity was defined as the EPC being valid (no more than 10 years old) at least on the first day of the data period. For SHM data, each period was considered separately. Thus, if an SHM has two periods, one period may have EPC information available, and the other may not, or the information may differ between the periods. In addition, several EPCs can be valid simultaneously, as the EPCs are not invalidated when a new EPC is issued. If two EPCs are valid for an SHM or SWM, the information from the most recent EPC was used. Furthermore, if an EPC was issued during the data period of the respective SHM or SWM, all EPCs were considered invalid for this period, as it is assumed that the building has been renovated and, therefore, the data represent two different building conditions. The need for this assumption also originated from the fact that it is currently not possible to easily track the changes from one EPC to another.

## Limitations

Despite the substantial efforts invested in mitigating the uncertainty associated with building characteristics data, it is important to acknowledge that some level of uncertainty persists. In addition, the BBR database used has no version control or modification history. Therefore, the data can only be extracted from the current version. Therefore, it cannot be ruled out that the data changed between the time the SHM or SHW data were recorded and the time the BBR data were retrieved.

## Ethics Statement

The authors have read the ethical requirements and confirm that the current work does not involve human subjects, animal experiments, or any data collected from social media platforms. The authors declare further that they obtained permission to use the primary data.

## CRediT authorship contribution statement

**Markus Schaffer:** Conceptualization, Methodology, Software, Formal analysis, Data curation, Writing – original draft, Writing – review & editing. **Martin Veit:** Methodology, Software, Data curation, Writing – review & editing. **Anna Marszal-Pomianowska:** Conceptualization, Methodology, Writing – review & editing, Supervision, Project administration, Funding acquisition. **Martin Frandsen:** Methodology, Writing – review & editing. **Michal Zbigniew Pomianowski:** Methodology, Writing – review & editing. **Emil Dichmann:** Software, Data curation, Writing – review & editing. **Christian Grau Sørensen:** Software, Data curation, Writing – review & editing. **Jesper Kragh:** Data curation, Writing – review & editing.

## Data Availability

Dataset of smart heat and water meter data with accompanying building characteristics (Original data) (AAU VBN)
